# Shoulder-Tip Pain in Hepatic Diseases

**Published:** 1871-02

**Authors:** 


### SHOULDER-TIP PAIN IN HEPATIC DISEASE.

In a most elaborate paper on the Shoulder-tip Pain and other Sympathetic Pains in Diseases of the Liver, read at the meeting of the British Medical Association in August last, by Dr. Embleton, the learned author advances a new explanation of the nervous connection between the liver and the top of the shoulder. First stating that according to his experience, the shoulder-pain occurs much more frequently than is supposed by some writers, he remarks that it is " referred especially to the angular space between the acromial end of the spine of the scapula and the adjacent end of the clavicle, where the upper and outer part of the trapezius muscle, as it goes to be inserted into the bones just named, is lodged ;" and adds :

"At this part, the external branch of the spinal accessory nerve, after having supplied the sterno-cleido-mastoid, and anastomosed with the second and third cervical nerves, enters the trapezius, forming a small plexus with twigs of the third and fourth cervical nerves, and is continued on along the vertebral border of the scapula, supplying the trapezius in its course, and communicating with twigs of the intercostal nerves."

It is noticed that this pain, when severe, extends from the shoulder-tip downwards to the lower angle of the scapula, and occasionally upwards along the side of the neck to the base of the skull, following the external branch of the spinal accessory nerve, which in such cases will be found tender to pressure, as will also the pneumogastric nerve of the same side. Pressure upon either of these nerves will sometimes aggravate the shoulder-tip pain, and, upon the vagus, will excite or increase pain in the liver.

"The course, distribution and office of the external divisions of the spinal accessory appear not to be in doubt. That the internal division joins the trunk of the parvagum at the ganglion of the trank, and contributes to the formation of the pharyngeal and laryngeal nerves with the vagus, is not doubtful; but we are ignorant as to how far the remaining part of the spinal accessory, which is incorporated with the vagus, goes along with it, and we do not know to which organs it is finally distributed. These two points are of necessity, from the nature of the parts, of most difficult, if not impossible, determination by the scalpel. By some anatomists, the spinal accessory portion of the vagus is believed to be given to the lungs, heart and stomach, which it is said to animate, more or less, with motor power (Hirschfeld and Leveille, *Nevrologie et Æsthesiologie*, 2me edition, Paris, 1866, p. 218 *et seq.*) If this be true, there seems to be no reason why the liver, kidneys and other parts—such as the supra-renal bodies, the pancreas and the small intestine—should not receive branches from the spinal accessory as well as from the vagus itself. In such case, we should have the seat of the sympathetic pain supplied by the external division, and the seat of the disease in the liver supplied, in part, at least, by the internal division of the same nerve—the spinal accessory—the two branches being in intimate connection beneath the skull. But, although it is probable that the internal branch of the spinal accessory is distributed to the same organs as the vagus itself, there are not as yet sufficient anatomical grounds for such a conclusion. It nevertheless appears that the par vagum, accompanied or not by the spinal accessory, does reach the liver, either directly from the gastric branches themselves, or indirectly from them through the great sympathetic ganglia; but the accounts of anatomists as to the mode and the amount of nervous supply to the liver, vary greatly from each other."

Numerous authors are cited to show the discrepancy referred to, though agreeing as to the main fact of the pneumogastric supplying the liver in some part at least.

The whole question of so-called sympathy between distant parts is one of much physiological interest, and every research is of value which tends to remove it from the domain of "vital" mystery and to confirm the law that sympathetic pain indicates nervous continuity.—*New York Med. Gazette.*

